# Omalizumab for chronic urticaria in Latin America

**DOI:** 10.1186/s40413-016-0127-y

**Published:** 2016-11-23

**Authors:** Paul Wilches, Paola Wilches, Juan Carlos Calderon, Annia Cherrez, Ivan Cherrez Ojeda

**Affiliations:** 1 Allergy Department, Hospital Monte Sinai, Cuenca, Ecuador; 2 Universidad Estatal de Cuenca, School of Medicine, Cuenca, Ecuador; 3 Universidad de Especialidades Espíritu Santo, School of Medicine, Samborondón, Guayas 09150 Ecuador; 4 Respiralab Research Group, Guayaquil, Ecuador; 5 University of Heidelberg, School of Medicine, Heidelberg, Germany

**Keywords:** Omalizumab, Chronic urticaria, Anti-IgE, Real life study, Latin America

## Abstract

**Background:**

Chronic urticaria (CU) is defined as the spontaneous appearance of wheals, with or without angioedema, persisting for ≥6 weeks. Chronic Spontaneous Urticaria (CSU) is a type of CU which affects 0.5–1 % of the global population, but it represents a high burden to patients. In recent years, omalizumab is available as treatment of disease. Our aim is to extend previous findings, analyzing effects of omalizumab on symptoms in Latin American patients with CSU.

**Methods:**

Retrospective analysis of patients treated with omalizumab in Cuenca-Ecuador. 150 mg omalizumab was administered every 4 weeks, and its effects were measured by Urticaria Activity Score (UAS) at baseline and each month in follow up. Complete response was defined as a UAS of 0 or 1, and partial response was classified as a UAS of 2 or more. Also, demographic and clinical variables were collected. Descriptive analyses were employed. Response rates were summarized as counts and percentages after 3 and 5 months. Related Samples Wilcoxon signed rank tests were used to compare UAS at baseline and after 3 months. *P* values <0.05 indicated statistical significance.

**Results:**

26 subjects were enrolled, almost half were female individuals (57.7 %), with mean age 47.8 years (range, 18–81 years). Mean duration of CU after diagnosis was 23.3 months (range, 2–180 months). Mean UAS at baseline was 5.7 points (range, 4–6 points). Nine patients (34.6 %) completed 3 months of treatment (33 % reported a complete response), with a mean difference in UAS of 3.33 (*p* = 0.01). Four patients completed 5 months of treatment (75.0 % showed a complete response). All patients previously treated with first-generation antihistamines plus corticosteroids showed no responses at neither 3 nor 5 months of treatment.

**Conclusion:**

Omalizumab is an effective treatment for patients with CU. It is necessary to conduct some future investigations where we can establish if 150 mg could be an option in developing countries.

**Electronic supplementary material:**

The online version of this article (doi:10.1186/s40413-016-0127-y) contains supplementary material, which is available to authorized users.

## Background

Urticaria is a disease characterized by the development of wheals (hives) and/or angioedema. Chronic urticaria (CU) is defined as the spontaneous appearance of wheals, with or without angioedema, persisting for ≥6 weeks. CU has been categorized into two main types: chronic spontaneous urticaria (CSU) owing to known or unknown causes and inducible urticaria (CIndU) [1]. CSU has been reported to affect 0.5–1 % of the global population at any given time and to account for approximately two-thirds of all cases of CU. CSU can have a considerable burden on patients, healthcare systems, and society [2].

Studies evaluating the economic impact of CSU have estimated that the total annual cost per patient in the US is $2047, with indirect costs accounting for 15.7 % ($322) [3]. This disease has been associated with many daily activities and can affect quality of life, for example, by contributing to absence from work or impairing work performance [3, 4].

Although antihistamines are the mainstay of treatment for CSU, some patients are non-responsive to higher doses of H_1_-antihistamines. Treatment guidelines for these patients recommend the addition of omalizumab, cyclosporine, or montelukast [1].

Omalizumab is a humanized monoclonal anti-immunoglobulin E (IgE) antibody, at a dose of 150 and/or 300 mg every 4 weeks for 3 or 6 month showed a significant improvement of itching score and UAS 7, with a sustained control of CSU symptoms in clinical trials [5–7]. This agent also showed good efficacy in real-life studies, in that approximately 82 % of patients had a complete or significant response to 150 or 300 mg omalizumab every 4 weeks, and 60 % stopped taking concomitant medications; moreover, omalizumab is well tolerated and has a good safety profile [8, 9]. Furthermore, excellent rates of response using adjusted [10, 11], as well as fixed omalizumab doses were observed in CSU patients [12].

To extend previous findings, this retrospective study analyzed the effects of omalizumab on symptoms in Latin American patients with CSU.

## Methods

This retrospective analysis involved 26 patients who presented with anti-histamine refractory CSU from January 2012 to April 2015 and were treated with omalizumab in Cuenca-Ecuador. Anti-histamine-refractory CSU was defined as having uncontrolled symptoms (defined as persisting of pruritus and urticaria) for at least 6 weeks, despite an up to 4-fold increase in the dose of non-sedating H_1_-antihistamine and/or the addition of other therapeutic agents, such as H_2_or sedating antihistamine, for 4 weeks [1]. Patients were subcutaneously administered 150 mg omalizumab every 4 weeks, with the dose being independent of patient body weight and serum IgE level.

The effects of omalizumab were analyzed by measuring the Urticaria Activity Score (UAS), a validated measure assessing disease activity in patients with CIU/CSU, before and after treatment by the patient and with supervising of the same physician or assistant physician in each visit [13]. The UAS evaluates itch severity and number of hives daily, with 0–3 points for each and total scores of 0–6 [13]. Complete response was defined as the disappearance of hives and pruritus, with a UAS of 0 or 1, and partial response was classified as a UAS of 2. Patients were classified as non responders when neither remission nor any improvement in symptoms was experienced during the treatment period or the UAS was >2 after3 and 5 months of treatment. Reduction of concomitant medication after three and five omalizumab doses was assessed as secondary outcome during follow-up. The study was approved by the Ethics Committee of Hospital Monte Sinaí in Cuenca, Ecuador.

Demographic and clinical variables were also collected, including age, sex, and previous medications. Medications were subclassified as first and second generations of anti-histamines and corticosteroids, alone or in combination. Other variables evaluated included duration of CSU and IgE levels.

### Statistical analysis

Descriptive analyses (frequency and percentage, standard deviation and range) were employed for demographical and clinical variables at baseline and follow-up. Response rates were summarized as counts and percentages after 3 and 5 months. Normal distribution of data was assessed by the Lilliefors corrected Kolmogorov-Smirnov test. Related Samples Wilcoxon signed rank tests were used to compare UAS at baseline and after 3 months and 5 months. Post hoc backward regression analyses were performed in order to identify responders to low dose/short period of treatment with omalizumab. All statistical analyses were performed using SPSS software version 19.0 (SPSS, Inc., Chicago, IL, USA), with *P* values <0.05 indicating statistical significance.

## Results

The 26 subjects enrolled in this study included 15 female individuals (57.7 %) and 11 male individuals (42.3 %), of mean age 47.8 years (range, 18–81 years). Mean duration of CU after diagnosis was 23.3 months (range, 2–180 months) and the mean IgE level was 570.6 kU/L (range, 55–2500 kU/L). Six patients (23.1 %) had comorbidities, and thyroid disease was included in the differential diagnosis of ten (38.5 %) patients (Table 1).

**Table 1:** Patient characteristics at baseline

Characteristic	
Mean age, years (range)	47.8 (18–81)
Sex: female, *n* (%)	15 (57.7)
Mean duration of CU, months (range)	23.3 (2–180)
Mean IgE, kU/L (range)	570.6 (55–2500)
Differential diagnosis of thyroid disease, *n* (%)	10 (38.5)
Comorbidities, *n* (%)	6 (23.1)
Previous medication, *n* (%)	26 (100)
-First-generation antihistamines alone	5 (19.2)
-Second-generation antihistamines alone	6 (23.1)
-First-generation antihistamines plus corticosteroids	3 (11.5)
-Second-generation antihistamines plus corticosteroids	11 (42.3)
-First- and second-generation antihistamines	1 (3.8)

*CU* chronic urticaria, *IgE* immunoglobulin E

At initiation of omalizumab treatment, the mean UAS in the 26 patients was 5.7 points (range, 4–6 points) (Table 2). All 26 patients had been treated previously: 11 (42.3 %) administered second-generation antihistamines plus corticosteroids and six (23.1 %) administered second-generation antihistamines alone (Table 1). Among patients receiving corticosteroids plus antihistamines, in average it was administered corticosteroids among 3–4 months, and even one of them was using intramuscular corticosteroids once a month by 6 months plus hydroxyzine by 2 years. Patients who had been receiving second generation antihistamines, they were receiving a two-fold an even four-fold increased doses in an average of 3 months. Among five patients taking first generation antihistamines, all of them were administered with hydroxyzine (25–50 mg) in an average of 2 months.

**Table 2:** Mean UAS throughout treatment

Time (months)	No. of patients	Mean UAS (SD)
Baseline	26	5.6 (0.6)
1	26	3.4 (1.3)
2	13	2.7 (1.5)
3	9	2.3 (1.7)
4	6	1.3 (0.8)
5	4	2.0 (2.0)

*UAS* urticaria activity score

Of the 26 patients, nine (34.6 %) completed 3 months of omalizumab treatment, whereas 17 (65.4 %) didn’t.

**Fig. 1:**
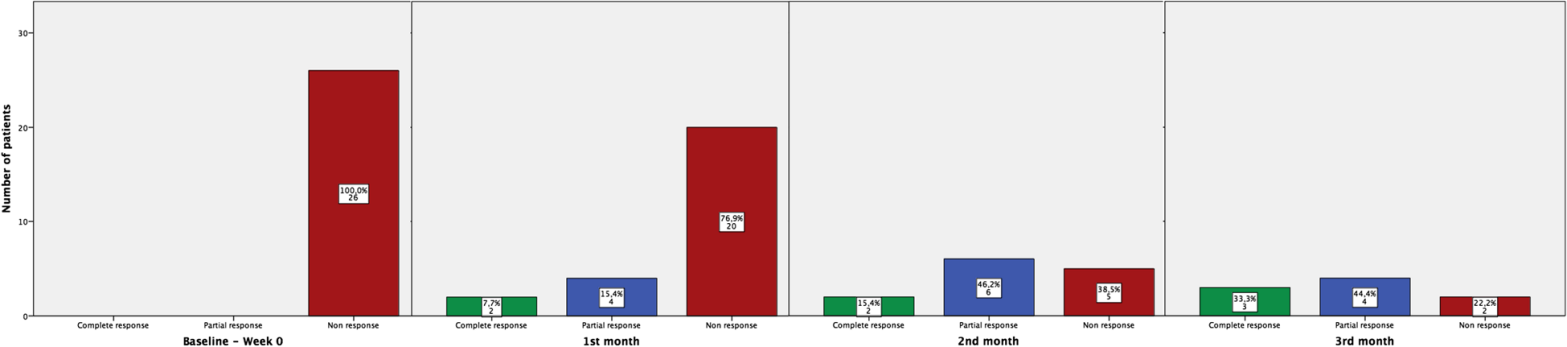
Frequencies of complete (*green*), partial (*blue*) and non responses (*red*) to 150 mg of omalizumab

Over time, UAS decreased from a mean 5.7 points to a mean 2.0 points (Table 2). Of the nine patients who completed 3 months of omalizumab treatment, three (33 %) showed a complete response (UAS of 0–1 point) and four (44.4 %) showed a partial response (UAS of 2 points) (Fig. 1). These nine patients had a mean UAS at 3 months of 2.3 (SD 1.7) points (Table 2). Compared with the mean UAS at baseline of 5.7 (SD 0.7) points in the 26 patients, the mean difference between baseline and 3-month UAS (*n* = 9) was 3.3 points (95 % confidence interval, 2.0–4.7 points, *p* = 0.01). Of the four patients who completed 5 months of treatment, three (75.0 %) showed a complete response (Table 3), with a mean difference between baseline and 5-month UAS of 4.0 (95 % CI 0.8–7.2). No serious adverse events were reported.

**Table 3:** Rate of response after 3 and 5 months, *n* (%)

Time (months)	No. of patients	Complete response	Partial response	Non-response
3	9	3 (33.3)	4 (44.4)	2 (22.2)
5	4	3 (75.0)	0 (0.0)	1 (25.0)

Patients who received or not the three doses were similar in response at the beginning of treatment. Nine patients who received third doses began with a UAS of 5.7 (SD 0.7). After, they continued with a UAS of 3.1 (SD 1.4) at first month, and UAS of 2.6 (SD 1.6) at second month of treatment. Meanwhile, patients who didn’t receive the third doses began with a similar UAS (5.6, SD 0.5), and after one (3.5, SD 1.3) and second month of treatment (3.0, SD 1.4) remained similar.

Of the 17 patients (65.4 % of all patients) who didn’t receive a third doses: 23.5 % (four patients) received two doses and 76.5 % (13 patients) only received one dose. Among patients who received two doses, half reported UAS of two (partial response), one reported a UAS of 3 and another reported a UAS of five (non responders). Patients with partial response didn’t comeback, and they reported relieving of symptoms when were contacted by phone. In non responders, one patient complained about high cost of treatment and the other was a lost of follow-up.

Between patients who only received first doses (13 subjects), two (15.4 %) reported complete response (one patient complained about high cost of treatment). In the 11 remaining patients, mean UAS was 3.8 (SD, 0.6). In this group of patients, complaints about high cost of treatment was 63.6 %, and remaining patients were lost of follow up.

Of three patients who achieved a complete response at fifth month of treatment, three of them reported complete and only one reported partial response at third month. But, all of them reported complete response at fourth month.

Although UAS at 3 and 5 months did not correlate significantly with previous medications (p > 0.05), all patients previously treated with first-generation antihistamines plus corticosteroids showed no responses at three (mean UAS 3.5, range 2–6) and five (mean UAS 3, range 1–5) months.

Finally, all patients were discontinued of administering corticoids during all follow up. After first administration, 65.4 % (*n* = 17) patients were administering levocetirizine 5 mg, 11.1 % (*n* = 3) were taking levocetirizine 10 mg, and 11.1 % were taking hydroxyzine 10 mg. Also, 3.7 % (*n* = 1) were taking hydroxyzine 25 mg, 3.7 % were taking levocetirizine 5 mg plus hydroxyzine 10 mg, and 3.7 % loratadine 10 mg plus hydroxyzine 50 mg.

After third administration, one of the patient (50.0 %) taking levocetirizine 10 mg, diminished the dose to 5 mg of levocetirizine. Otherwise, one of the patient (20.0 %) taking levocetirizine 5 mg discontinued this medication after receive 5 months of omalizumab.

The patient under loratadine 10 mg plus hydroxyzine 50 mg at the beginning of this study, he diminished dose of hydroxyzine to 25 mg but continued taking loratadine 10 mg after fifth administration of omalizumab (﻿Aditional ﻿file 1: Database file).

## Discussion

Large multicenter, randomized, double-blind, placebo-controlled phase III trials have shown that omalizumab, at doses of 150 and 300 mg every 4 weeks for 3 months, significantly improved urticaria outcomes compared with placebo in patients with CSU [5–7]. Moreover, broader studies in actual clinical practice have provided strong evidence for the efficacy of omalizumab, showing a complete or significant response in approximately 82 % of patients, with 60 % of patients stopping concomitant medications [8].

One of the limitations of retrospective studies is their use of subjective methods of evaluation to show categorical responses to omalizumab [8, 14, 15]. A study from Spain, in which the UAS was used to assess disease activity, found a significant reduction in the mean UAS in a subgroup of 38 patients, from 5.34 ± 0.88 before treatment to 0.66 ± 1.3 after 3 months (*p* < 0.005) [8]. Similarly, using UAS, we found that 77 % of our patients had a complete or partial response after treatment with omalizumab for 3 months, and a recent study reported that 60 % of patients responded when started on the same doses [14]. The use of concomitant medication significantly decreased, because 60 % of patients were able to withdraw all medications using omalizumab [8]. In our patient after 3 doses we reduce concomitant medication in one patient (11.1 %), and withdraw all medications in another one patient (11.1 %). After five doses, the reduction of concomitant medication was reported by one patient (25 %) and withdraw in another patient (25 %).

However, 65.4 % of patients didn’t complete 3 months of treatment, most of them (47.1 %) because the cost of omalizumab and it not being reimbursed by health insurance programs. Kaplan et al. [16] established that they are not aware of any formal definition of response to treatment in patients with CIndU/CSU. Uysal P et al. [17] found that 55.5 % of patients reached a UAS < 2 after two or three doses of 150 mg of omalizumab, and no definition of response to treatment was stablished. Recently, Palacios T et al. [18] found that basophil CD203c-upregulating activity could predict lower clinical response, but further studies are needed to confirm this association. In our study, we used post-hoc backward logistic regression analyses in order to identify responders to low dose/short period of treatment with omalizumab. It was included sex, age, time with disease, previous medication, and UAS at first month and third month. Any factor was identified as marker of response to treatment after third or fifth doses of omalizumab. It could be explained because small sample size and lack of power. It is likely that patients who did not response to monthly dose of 150 mg, they would had responded to doses of omalizumab of 300 mg.

In countries where economic issues make access to treatment difficult, a monthly dose of 150 mg should be a good option to control CU. In our not responder patients, one options could be increase dose to 300 mg. Further research about timing of response, length of treatment, delay in response and identifying specific phenotypes in response to omalizumab would be essential to physicians who have patients requiring omalizumab for management of CSU. Also, it is necessary to conduct some future investigations where we can establish if 150 mg could be an option in developing countries. No adverse effects were reported in any patient during treatment, confirming the long-term safety profile of omalizumab.

## Conclusions

CU can have a negative impact on the physical, emotional and social lives of patients. Omalizumab represents a new and effective treatment for patients with CU. Treatment of patients with low incomes should be individualized because the high cost of this agent prevents patients from completing at least 3 months of treatment. It is necessary to conduct some future investigations where we can establish if a lower dose of omalizumab (150 mg) could be an option in low/middle income countries.

## Additional file

Additional file 1: Database file. (SAV 17 kb)

## Abbreviations

CIndU: Chronic inducible urticaria
CSU: Chronic spontaneous urticaria
CU: Chronic urticaria
UAS: Urticaria activity score
